# Proscillaridin A is cytotoxic for glioblastoma cell lines and controls tumor xenograft growth *in vivo*

**DOI:** 10.18632/oncotarget.2541

**Published:** 2014-10-21

**Authors:** Emilie Denicolaï, Nathalie Baeza-Kallee, Aurélie Tchoghandjian, Manon Carré, Carole Colin, Carine Jiguet Jiglaire, Sandy Mercurio, Christophe Beclin, Dominique Figarella-Branger

**Affiliations:** ^1^ Inserm, Aix-Marseille Université, CRO2 UMR_S 911, Marseille, 13385, France; ^2^ CNRS, IBDML, Aix Marseille Université, UMR_S 6216, Marseille, 13288, France; ^3^ APHM, Hôpital de la Timone, Service d'Anatomie Pathologique et de Neuropathologie, Marseille, 13385, France

**Keywords:** Proscillaridin A, glioblastoma, cytotoxicity, tumor growth control, Na^+^/K^+^ ATPase α1 subunit

## Abstract

Glioblastoma is the most frequent primary brain tumor in adults. Because of molecular and cellular heterogeneity, high proliferation rate and significant invasive ability, prognosis of patients is poor. Recent therapeutic advances increased median overall survival but tumor recurrence remains inevitable. In this context, we used a high throughput screening approach to bring out novel compounds with anti-proliferative and anti-migratory properties for glioblastoma treatment. Screening of the Prestwick chemical library® of 1120 molecules identified proscillaridin A, a cardiac glycoside inhibitor of the Na^+^/K^+^ ATPase pump, with most significant effects on glioblastoma cell lines. *In vitro* effects of proscillaridin A were evaluated on GBM6 and GBM9 stem-like cell lines and on U87-MG and U251-MG cell lines. We showed that proscillaridin A displayed cytotoxic properties, triggered cell death, induced G_2_/M phase blockade in all the glioblastoma cell lines and impaired GBM stem self-renewal capacity even at low concentrations. Heterotopic and orthotopic xenotransplantations were used to confirm *in vivo* anticancer effects of proscillaridin A that both controls xenograft growth and improves mice survival. Altogether, results suggest that proscillaridin A is a promising candidate as cancer therapies in glioblastoma. This sustains previous reports showing that cardiac glycosides act as anticancer drugs in other cancers.

## INTRODUCTION

Gliomas are the most common primary brain tumors in adults [1]. Among them, glioblastomas (GBM) represent the ultimate grade of malignancy and is one of the most aggressive cancers with a median overall survival (OS) of about 15 months after surgical resection followed by radiotherapy and temozolomide (concomitant then adjuvant) which is the standard of care [2]. More recently, two larger randomized phase III trials (AVAglio and RTOG 0825), which aimed to compare anti-angiogenic therapy (bevacizumab) with standard of care, failed to demonstrate significant OS improvement [3]. Therefore, GBM remain with poor prognosis and require new therapeutic approaches.

GBM show a high proliferation rate and invasive properties which are the major causes of recurrence. In addition, GBM are highly heterogeneous. Indeed, molecular characterization of these tumors allowed the identification of gene expression-based molecular classifications into four subtypes: proneural, neural, classical and mesenchymal, which seemed to present distinct outcome [4, 5]. GBM are also heterogeneous regarding treatment response and up to now the MGMT promoter methylation status remains the best biomarker for response to temozolomide [6]. At last, heterogeneity is also recorded regarding cell composition, since GBM may derive from cancer stem cells that are mainly involved in gliomagenesis, treatment resistance, invasion and thus tumor recurrence [7–11]. Therefore, identification of new therapies should take into account GBM molecular and cellular heterogeneity.

In order to find novel anticancer compounds, we have first screened the Prestwick chemical library® to find molecules with anti-proliferative and anti-migratory properties towards two human primary GBM stem cell lines, GBM6 and GBM9, previously established and characterized in our laboratory [11, 12]. They represent two appropriate study models of GBM that differed in their cellular composition, displayed specific molecular signature and strikingly differed in their *in vitro* behavior. Indeed, GBM6 cells were highly migratory whereas GBM9 cells exhibited much lower migratory capacities but a higher proliferation rate. Moreover, they displayed distinct *in vivo* growth pattern [11, 12]. The screening allowed to select the best candidate molecule, proscillaridin A, a cardiac glycoside inhibitor of the Na^+^/K^+^ adenosine triphosphate (ATPase) pump. Na^+^/K^+^ ATPase is a transmembrane protein that catalyzes the active transport of Na^+^ and K^+^. In addition, it may participate to signal transduction complexes [13]. Structurally, Na^+^/K^+^ ATPase is a heterodimer of a catalytic α-subunit and a glycosylated β-subunit. The α-subunit has binding sites for ATP, Na^+^, K^+^ and cardiac glycosides. Literature reported that α1 subunit was preferentially expressed in glial cells [14] and that an increase in the α1 subunit level enhanced sensitivity to cardiac glycosides [15]. Here, we analyzed the expression of Na^+^/K^+^ ATPase α1 subunit, known as the putative target of proscillaridin A, in a series of GBM human samples and we confirmed its expression in our GBM cell line models. In a second step, we analyzed the *in vitro* and *in vivo* effects of proscillaridin A. We showed that proscillaridin A induced apoptosis, decreased cell proliferation by blocking cell cycle at the G_2_/M phase in all GBM cell lines. In addition, proscillaridin A impaired *in vitro* GBM stem cells self-renewal capacity, reduced tumor growth and improved mice survival *in vivo*.

## RESULTS

### Prestwick chemical library® *in vitro* screening

**Figure 1:**
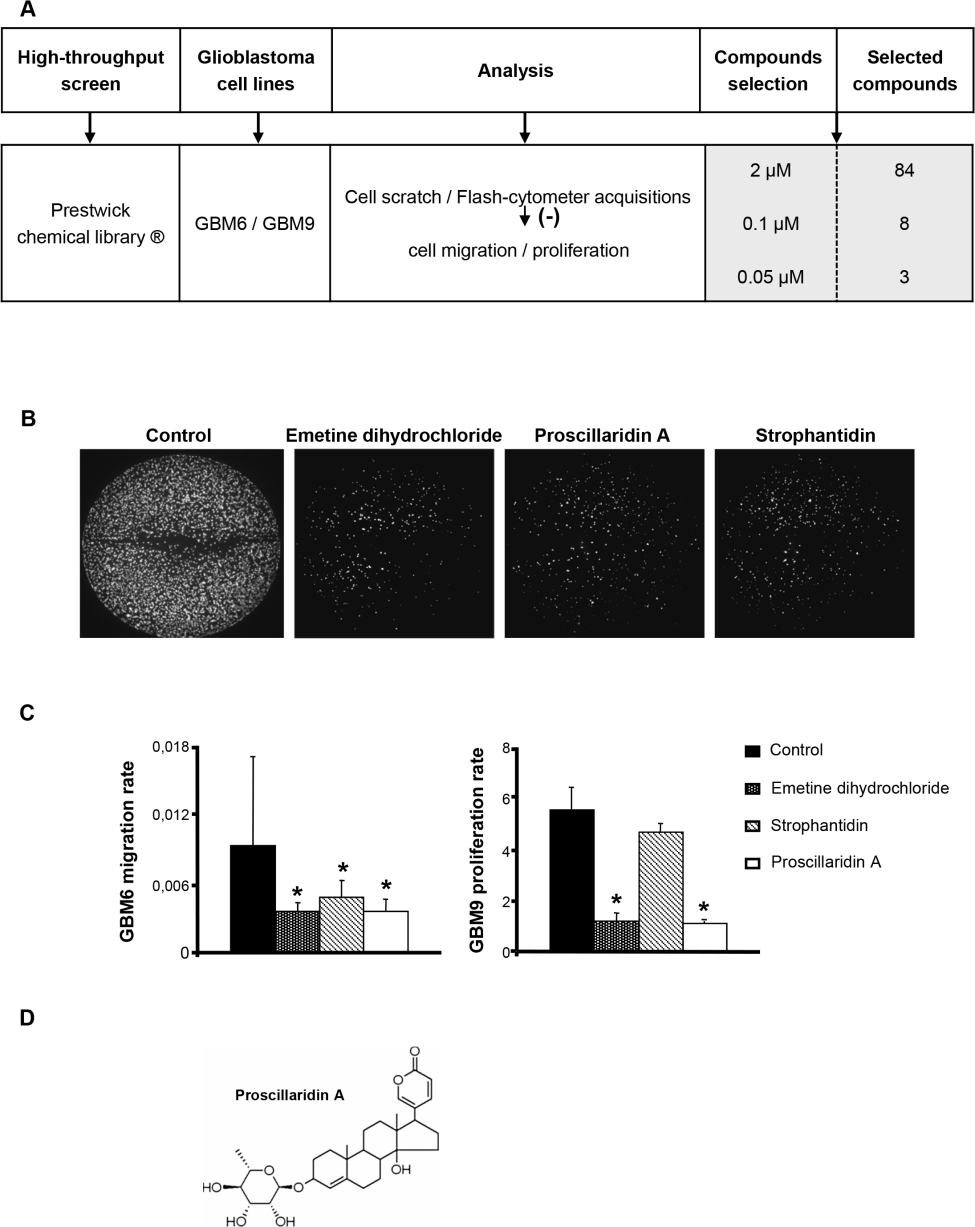
High-throughput screening of the Prestwick chemical library® on GBM cell lines and selection of proscillaridin A (**A**) The 1120 molecules of the Prestwick chemical library® were screened on GBM6 and GBM9 cell lines and selected according to their inhibitory efficacy on cell migration and proliferation. Effect was analyzed and quantified by cell scratch and flash-cytometer acquisitions. At 2 μM, 84 molecules were selected. At 0.1 μM, among the 84 molecules, 8 had a significant effect on cell migration and proliferation. At last, 3 molecules that exhibited a significant effect at 0.05 μM were selected. (**B**) GBM6 cells in 96-well black plates after cell scratch and 3 days of treatment with the 3 compounds selected, emetine dihydrochloride, proscillaridin A and strophantidin at a concentration of 0.1 μM. (**C**) Analysis of GBM6 migration and GBM9 proliferation rates after 3 days of treatment with emetine dihydrochloride, proscillaridin A and strophantidin at a concentration of 0.05 μM. Bars indicate the Standard Error of the Mean (SEM), *p<0.05, compared to DMSO control cells. (**D**) Chemical structure of proscillaridin A.

A synthesis of the procedure is summarized in Fig. 1A. The 1120 molecules were first tested at 2 μM and results showed that only 84 had a significant effect on both migration and proliferation of GBM6 and GBM9 cells. These 84 molecules were then tested at 0.1 μM and 8 molecules remained significantly efficient at this concentration. Two of them were anti-mitotic agents (paclitaxel, colchicin), one was a topoisomerase I inhibitor (camptothecin), one was a protein synthesis inhibitor (emetine dihydrochloride) and 4 were cardiotonic agents (strophantin octahydrate, digoxin, proscillaridin A and strophantidin) (data not shown). To better select the candidates, the 8 molecules were tested at 0.05 μM. Emetine dihydrochloride, proscillaridin A and strophantidin showed the most significant effect on both GBM6 migration and GBM9 proliferation rates (*p*<0.05) (Fig. 1B and C). According to these results and to previous relevant studies [16-18], we chose to further analyze only proscillaridin A (Fig. 1D) *in vitro* as well as *in vivo* on GBM tumor growth.

### Expression of Na^+^/K^+^ ATPase α1 subunit (*ATP1A1*)

**Figure 2:**
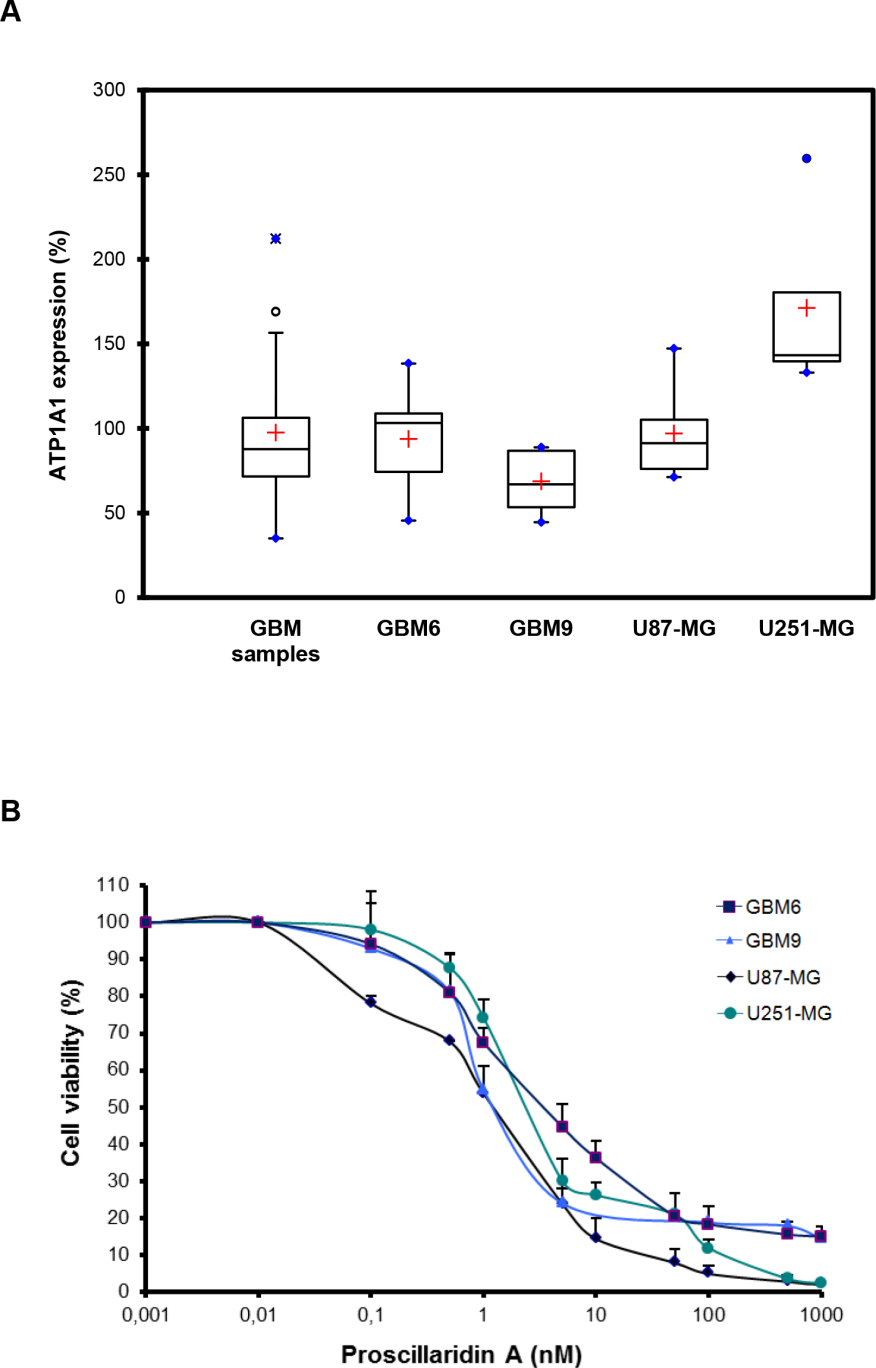
Evidence of *ATP1A1* in GBM human samples and cell lines and cytotoxic effect of proscillaridin A (**A**) Box plots of *ATP1A1* expression in GBM samples, GBM6, GBM9, U87-MG and U251-MG cell lines. The lower and upper edges of the box represent the first and third quartile respectively, while a horizontal line within the box indicates the median. The vertical length of the box represents the Interquartil range (IQR). The most extreme sample values (within a distance of 1.5 IQR from the median) are the endpoints of the lines extending from the box, while the black circles and the blue circles represent the outliers (1.5 IQR above 75th percentile) and far outliers (3 IQR above 75th percentile) respectively. a.u.: arbitrary unit. (**B**) Cells were treated for 72h with a concentration range (0 nM – 1000 nM) of proscillaridin A or DMSO. Cell viability was analyzed by MTT assay and is expressed as fold increase of DMSO controls.

First, we measured by real-time quantitative PCR (RT-qPCR) the expression of *ATP1A1* in a series of frozen human GBM samples (n = 26). The median expression level was 87.8 (IQR [71.7 – 106.3]) showing a widespread expression in the series despite an inter-individual variability (Fig. 2A). Prior to analyzing the *in vitro* and *in vivo* anti-tumoral effects of proscillaridin A on our GBM models we also analyzed the expression of *ATP1A1* in GBM6, GBM9, U87-MG and U251-MG cell lines. The median expression level was 103.20 (IQR [74.32 – 108.86]), 66.95 (IQR [53.45 – 86.85]), 91.26 (IQR [76.06 – 105.20]) and 143.31 (IQR [139.65 – 180.41]) respectively (Fig. 2A). We confirmed that the *ATP1A1* was expressed in all GBM cell lines with expression variations from one cell line to another in accordance to GBM samples results. Therefore, these 4 GBM cell lines represent appropriate models for the study of anti-tumoral effect of proscillaridin A on GBM.

### Cytotoxic effect of proscillaridin A and consequences on cell cycle

We evaluated the cytotoxic effect of proscillaridin A on GBM6, GBM9, U87-MG and U251-MG GBM cell lines by MTT assay. As shown in Fig. 2B, the cardiac glycoside had a dose-dependent inhibitory effect on cell survival. IC_50_ (concentration required to inhibit 50% of cell viability) values were 4 +/− 6 nM for GBM6, 3 +/− 5 nM for GBM9, 2 +/− 4 nM for U87-MG and 3 +/− 5 nM for U251-MG. Interestingly, these values were much lower than reported, for example, in breast and pancreatic cancer cell lines [19, 20]. Moreover, cytotoxic efficacy of other cardiac glycosides has been reported in some GBM cell lines [21] and our present results show that proscillaridin A is the most efficient in GBM.

**Figure 3:**
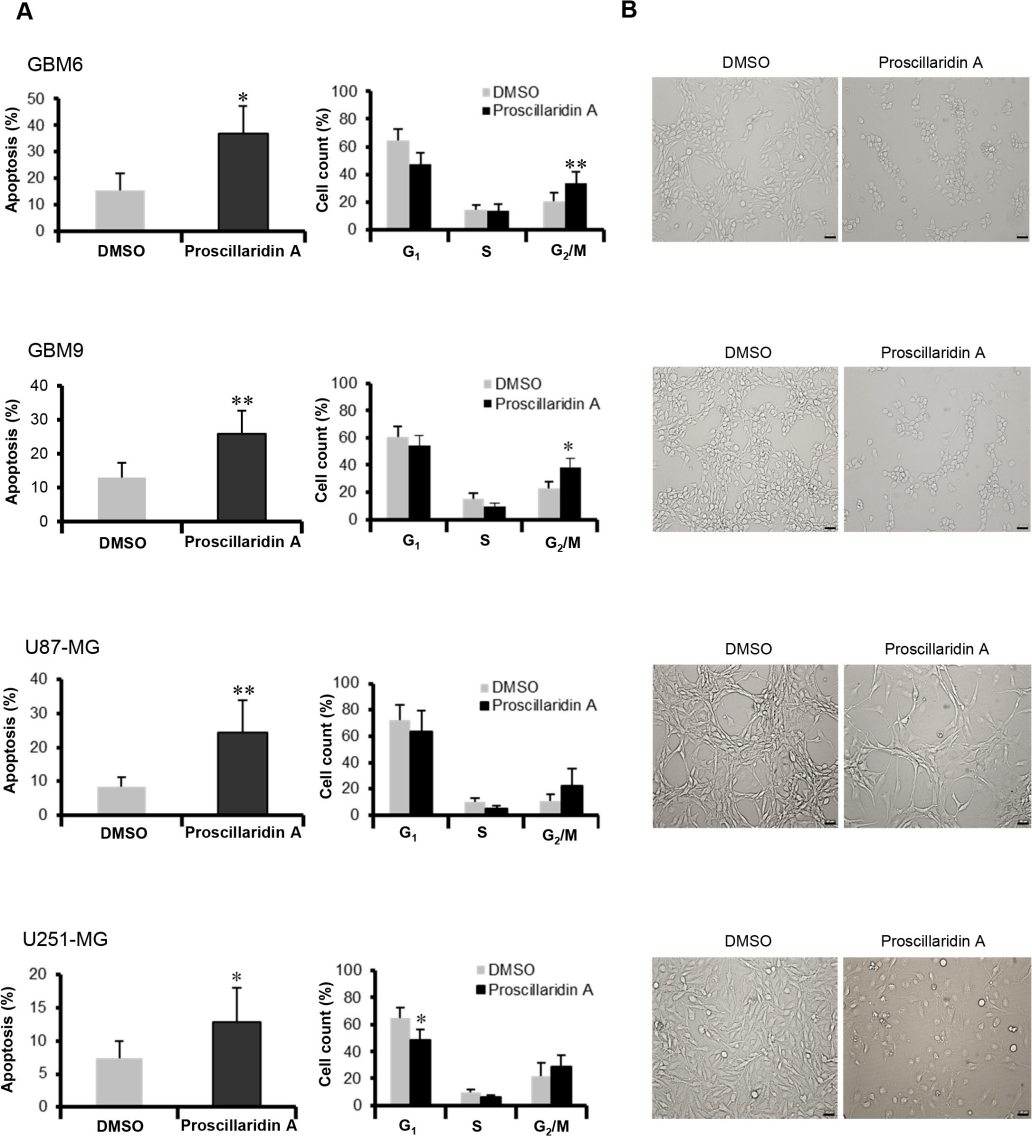
Apoptosis and cell cycle analyses (**A**) Apoptosis (Sub G_0_/G_1_) and cell cycle of DMSO control and proscillaridin A treated cells were determined by flow cytometry of propidium iodide-stained nuclei and percentage of apoptosis and cell count for G_1_, S and G_2_/M phases are shown. Data are expressed as mean ± SEM deduced from 5 independent experiments, *p<0.05 and **p<0.01 compared to DMSO control cells. (**B**) Morphology of cells treated for 72 hours with respective IC_50_ of proscillaridin A or DMSO. Scale bar: 40 μm.

To further characterize this cytotoxic efficacy, we measured cell death induction after a 72h treatment with the respective IC_50_ of proscillaridin A. Apoptotic (sub G_0_/G_1_) cells confirmed cell death with a significantly increase in GBM6 (*p*<0.05), GBM9 (*p*<0.01), U87-MG (*p*<0.01) and U251-MG (*p*<0.05) cell lines (Fig. 3A). Moreover, proscillaridin A similarly influences cell cycle progression in the 4 GBM cell lines (Fig. 3A). G_2_/M phase is increased in GBM6 (*p*<0.01) and GBM9 (*p*<0.05) cells and tends to be increased in U87-MG and U251-MG cells. G_1_ phase is decreased in U251-MG (*p*<0.05) cells and tends to be decreased for the 3 other cell lines. We did not find any significant effect on S phase. These results suggest that proscillaridin A (at IC_50_) blocks cell cycle at the G_2_/M phase and increases apoptotic process. As a result of these proliferation and survival inhibitory effects, the phenotype of GBM cell populations was highly modified, with a reduced cell density and a weakened cellular network (Fig. 3B).

### Proscillaridin A impairs GBM stem cell self-renewal capacity

**Figure 4:**
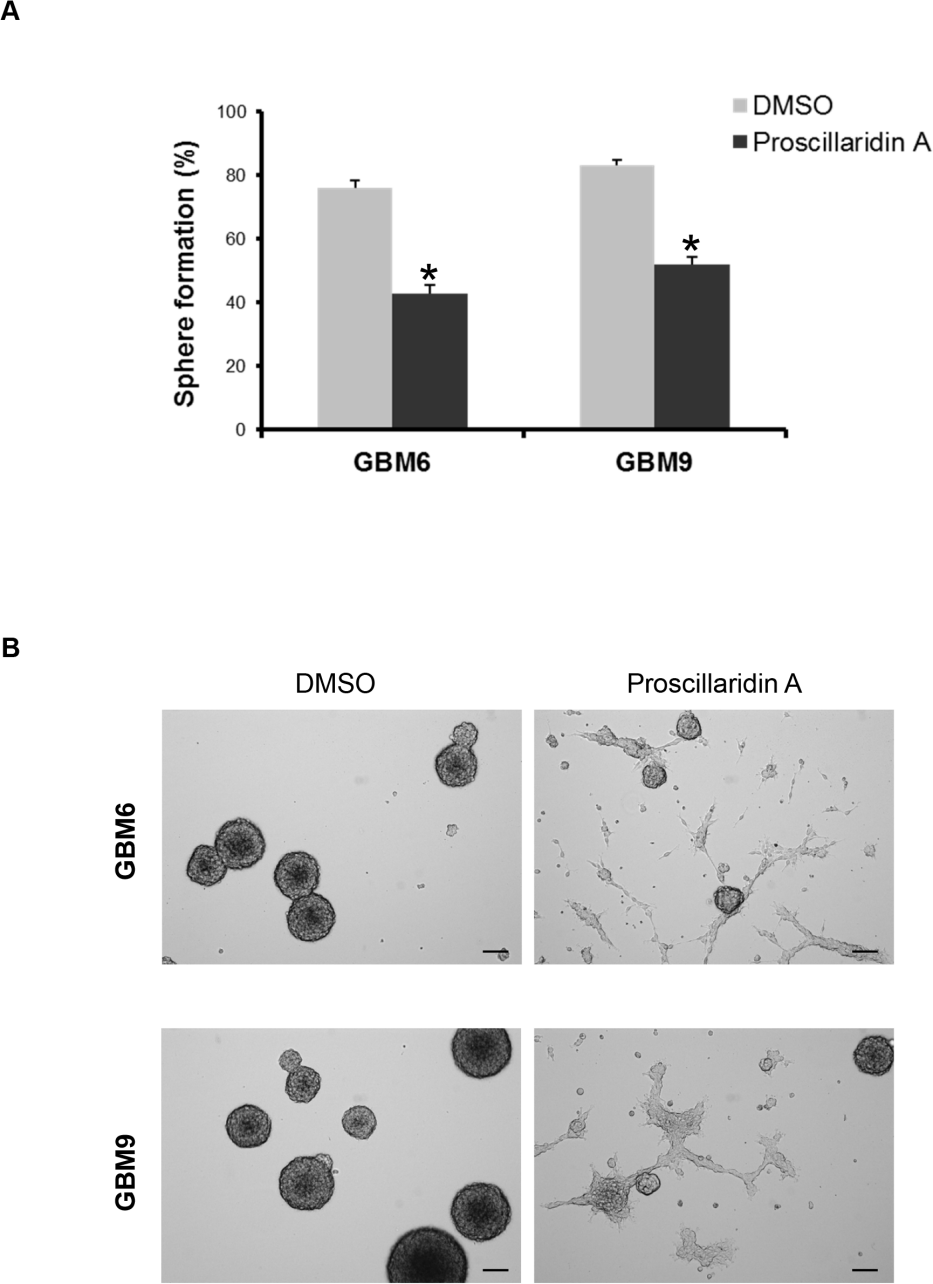
Proscillaridin A treatment and stem cell self-renewal (**A**) Self-renewal capacity of GBM6 and GBM9 stem cell lines was evaluated after 72h of treatment with respective IC_50_ of proscillaridin A or DMSO. The percentage of sphere formation was calculated as the number of spheres formed divided by the original number of cells seeded. Three experiments were performed in duplicate for each cell line. *p<0.05 compared to DMSO control cells. (**B**) Sphere assessment and cell morphology of GBM6 and GBM9 stem cell lines treated with DMSO or IC_50_ of proscillaridin A. Scale bar: 100 μm.

We evaluated the stem cell self-renewal capacity of GBM6 and GBM9 cells using limiting dilution. The quantification of self-renewal showed a significant decrease of sphere formation ability for both cell lines (*p*=0.002) (Fig. 4A). As a result of this impairment, many cells exhibited propensity to adhere to plastic plate. Consequently, GBM6 and GBM9 cell lines adopted a differentiation phenotype (Fig. 4B).

### Proscillaridin A reduces tumor growth *in vivo*

To highlight *in vivo* anti-tumoral effect of proscillaridin A on GBM development, we first achieved preliminary experiments on healthy *nude* mice in order to set up a treatment protocol. Briefly, daily doses (range: 0.5–20 mg/kg body weight) were evaluated for 7 days to exclude lethal doses. In this way, the dose of 7 mg/kg body weight was determined as the maximum tolerated dose in mice, and then kept for all the treatments applied in this study. At that dose, treatment was generally well tolerated as evidenced by an absence of weight loss or change in animal well-being as compared with vehicle (Dimethyl Sulfoxide (DMSO))-injected mice (data not shown).

**Figure 5:**
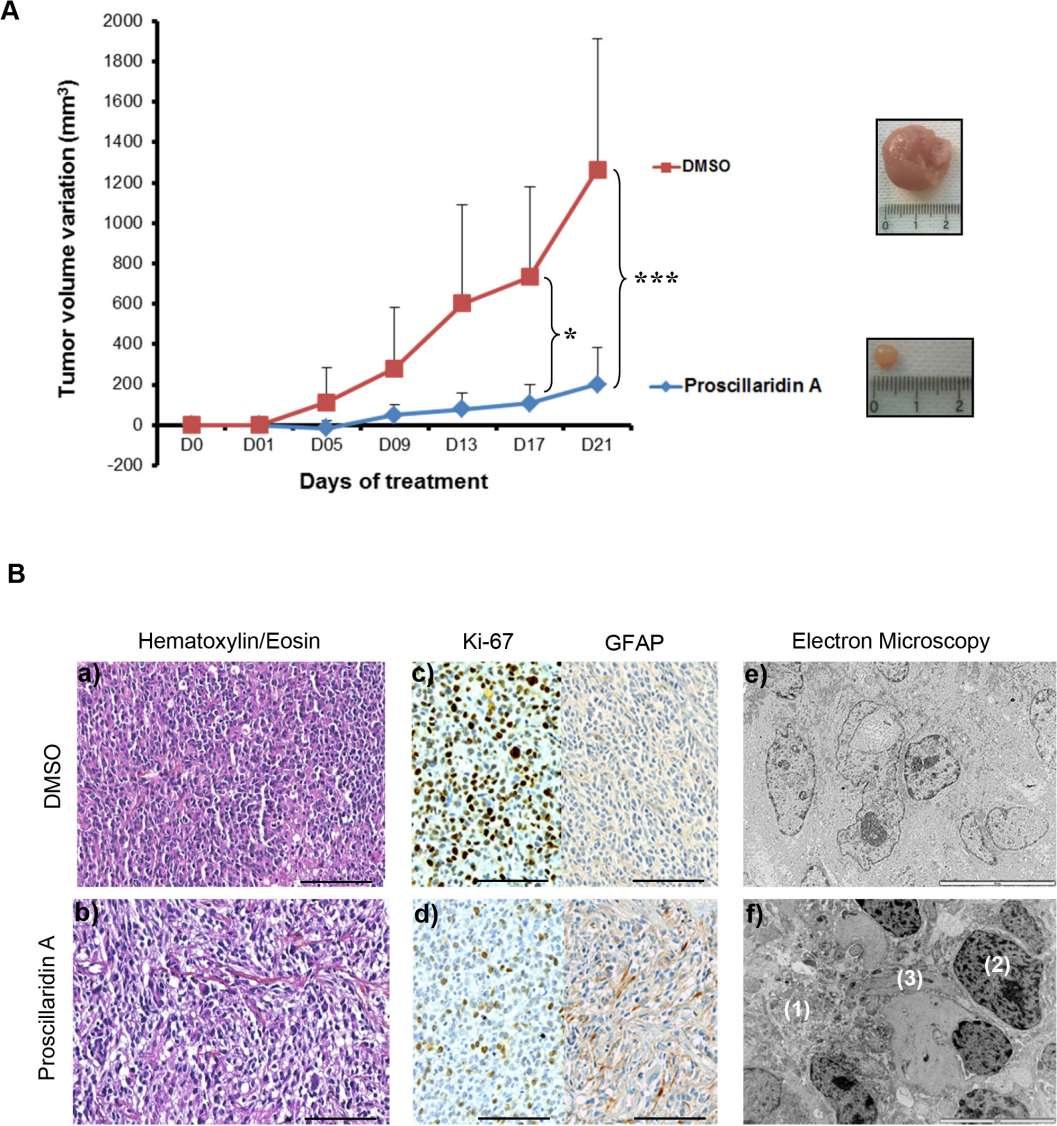
Proscillaridin A treatment and tumor xenograft growth (**A**) *Nude* mice were subcutaneously transplanted with 500 000 GBM6 tumor cells and randomized into 2 groups (n = 5 each) when tumors reached 2–4 mm^3^. Treatment consisted of intraperitoneal injection of proscillaridin A (7 mg/kg body weight) or vehicle, 5 days a week for a period of 3 weeks. Tumor volume was recorded every 4 days. Results show tumor volume variation (mm^3^) ± SEM. *p<0.05 and ***p<0.001. A representative picture of a tumor is shown as an inset. (**B**) Tumors were harvested on day 21 and tissues analyzed. Hematoxylin/eosin staining (a, b), Ki-67 staining for cell proliferation, GFAP staining for glial differentiation **(c, d)** and electron microscopy for tumor ultrastructure **(e, f)** were performed in control and proscillaridin A treated tumors: (1) enlarged intercellular space, (2) chromatin agregates in nucleus and (3) gliofilaments accumulation. Scale bar: 100 μm for hematoxylin/eosin staining and immunohistochemistry and 10 μm for electron microscopy.

For GBM6 subcutaneous tumors, 5 mice were treated with proscillaridin A and 5 control mice were injected with vehicle from 4 to 8 weeks after cells injection. Tumor volumes were recorded every 4 days (Fig. 5A). At day 5, a difference in tumor volume was already recorded. After 17 days, and even more after 21 days, the tumor volume was significantly decreased by proscillaridin A (*p*<0.05 and *p*<0.0001 respectively). At the end of the experiment, average tumor volume in control mice was 1266 mm^3^, while it was of 201 mm^3^ in treated mice. Tumors were sectioned and stained either with hematoxylin/eosin for conventional histology, or with Ki-67 or anti-Glial Fibrillary Acidic Protein (GFAP) antibodies. As expected, histological examination showed high cellularity in all GBM6 subcutaneous tumors. Enlarged intercellular spaces were observed in treated tumors, as a hallmark of proscillaridin A efficacy (Fig. 5B. a, b). Accordingly, GBM6 cell proliferation was slowed in treated tumors, as showed by the decrease in Ki-67 staining. Moreover, the increase in GFAP staining in proscillaridin A-treated tumors suggested GBM6 cell differentiation (Fig. 5B. c, d). Electron microscopy analyses also revealed differences between treated tumors and control ones (Fig. 5B. e, f). First, they confirmed the intercellular spaces enlargement in proscillaridin A-treated tumors (Fig. 5B. f(1)). In addition, treatment induced chromatin aggregation within nuclei (Fig. 5B. f(2)) and gliofilaments accumulation (Fig. 5B. f(3)), indicating ongoing cell death and differentiation respectively.

A total of 29 U87-MG subcutaneous tumors were also generated, to be treated with proscillaridin A (n = 15) or vehicle (n = 14), from 2 to 4 weeks after cells injection. At the end of the experiment, no significant difference was recorded between control and proscillaridin A treated-mice, suggesting that the tumors may escape from treatment probably due to high proliferation rate of U87-MG cells. New investigations with a different protocol of treatment (earlier starting time) need to be set up.

### Proscillaridin A improves survival of tumor-bearing mice

**Figure 6:**
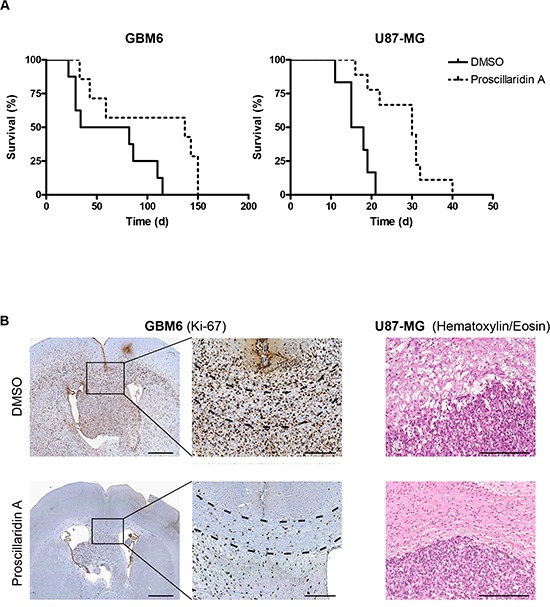
Efficacy of long term proscillaridin A treatment on survival of mice with intracranial tumor and histological analysis (**A**) Kaplan-Meier survival curves of mice intracerebrally grafted with GBM6 or U87-MG cell lines and treated with proscillaridin A. Time is expressed in days since start of treatment. (**B**) Ki-67 immunostaining of DMSO or proscillaridin A-treated GBM6 tumors. Full brain coronal section (left) and magnification showing infiltrating tumor cells within corpus callosum (dotted lines). Hematoxylin/eosin staining of DMSO or proscillaridin A-treated U87-MG tumors showing rim of tumors (right). Scale bar: 1 mm for full brain coronal sections and 250 μM for magnification and hematoxylin/eosin staining.

A total of 38 orthotopic intracranial tumors have been generated in *nude* mice after stereotactic injection of GBM6 (n = 21) or U87-MG (n = 17) cells. Among them, 11 mice (GBM6) and 10 mice (U87-MG) have been treated with proscillaridin A. The remaining mice (10 for GBM6 and 7 for U87-MG) were DMSO controls. At the end of the experiment, extracted brains have been sectioned, stained and microscopically analyzed for the presence of tumor. Tumors were recorded in 15/21 animals injected with GBM6 cell line (71%) and in 15/17 animals implanted with U87-MG cell line (88%). Only mice bearing tumors were included in survival analyses (Fig. 6A). Interestingly, proscillaridin A treatment induced significant survival benefits as compared to vehicle injection (*p*<0.05 and *p*<0.001 for GBM6 and U87-MG respectively). In GBM6 tumor-bearing mice, median survival was indeed more than two-times increased, from 58 days in control mice to 137 days in treated mice (Table 1). A similar improvement of median survival was measured in U87-MG bearing mice, from 16.5 days in control mice to 30 days in treated-ones (Table 2). For GBM6 tumors, strong differences were observed between control and treated mice. According to Ki-67 immunohistochemistry, we observed in control mice that cells migrated throughout the corpus callosum and used myelinated fibers to invade the brain giving rise to huge tumors. As expected, when treated with proscillaridin A, less GBM6 tumor cells were recorded and consequently they appeared as scattered in the corpus callosum (Fig. 6B). U87-MG tumors were histologically examined after hematoxylin/eosin staining. Tight tumors, sharply demarcated from the brain parenchyma, with irregular rim were recorded in 4/6 control brains as compared with 3/9 treated-ones, suggesting the anti-migratory property of proscillaridin A (Fig.6B).

**Table 1:** Median survival of treated mice with proscillaridin A as compared to control mice

GBM6	DMSO	Proscillaridin A
Mice	n = 7	n = 8
Median survival (days)	58	137
*p-value*	0,04	

**Table 2:** Median survival of treated mice with proscillaridin A as compared to control mice

U87-MG	DMSO	Proscillaridin A
Mice	n = 6	n = 9
Median survival (days)	16.50	30
*p-value*	0,001	

## DISCUSSION

In this study we have reported that proscillaridin A, a cardiac glycoside, is cytotoxic for GBM cell lines and controls tumor xenograft growth *in vivo*. Proscillaridin A was the best active compound selected after screening of the Prestwick chemical library® on GBM6 and GBM9 cell lines which represent two different appropriate models of human GBM [12]. Proscillaridin A exerts both anti-proliferative and anti-migratory activities in these cell lines at a concentration of 0.05 μM. According to these results and to previous relevant studies [16-18], we further analyzed only proscillaridin A *in vitro* as well as *in vivo* on GBM tumor growth. In addition to proscillaridin A, strophantidin, another cardiac glycoside and emetine dihydrochloride, a protein synthesis inhibitor, displayed similar properties, but their effects were less obvious. Interestingly, these compounds were much more active than temozolomide, the gold standard for GBM chemotherapy, which had no effect on migration nor proliferation rate of GBM6 and GBM9 cell lines at 0.1 and 2 μM.

Cardiac glycosides are a class of natural products. By increasing intracellular calcium, they increase cardiac contractile force in patients with congestive heart failure and cardiac arythmias [22–24]. Epidemiological studies first showed that digitalis could mediate anticancer effects: patients with breast cancer treated with digitalis have been found to have a lower death rate compared with those not treated [25]. A number of cardiac glycosides have demonstrated *in vitro* anti-tumor activity against a range of cancer types, including glioma [26–28]. Interestingly, cytotoxic profiles of seven cardiac glycosides in primary culture of tumor cells of different origins (leukaemia, renal cancer and myeloma) revealed that proscillaridin A was the most potent out of them [29].

We reported here that the four GBM cell lines studied, either growing as spheres (GBM6 and GBM9) or as adherent monolayer (U87-MG and U251-MG), were highly responsive to proscillaridin A. IC_50_ values indeed ranged from 2 to 4 nM, concentrations that are even lower than those used in other cell types studied [29]. Several mechanisms of cardiac glycosides have been hypothesized and the most widely accepted involves the ability of cardiac glycosides to bind to the membrane-bound Na^+^/K^+^ ATPase pump [30]. Accordingly to Lefranc *et al*. [15, 31], in the present study, RT-qPCR analysis of *ATP1A1* expression revealed that a series of GBM human samples and our GBM cell lines models commonly expressed the potential target, although expression level appeared heterogeneous in tumors as well as in cell lines. Inhibition of the ion pump activity by cardiac glycosides has been described to cause an increase in intracellular sodium concentration and a decrease in intracellular potassium concentration [30]. Moreover, besides its ion pumping activities, it is now established that the Na^+^/K^+^ ATPase acts as a scaffold for the assembly of a multiple protein signaling complexe in caveolae that transmits signal to various intracellular compartments [16, 32, 33]. Many different pathways have been suggested to be responsible for mediating cytotoxic effects of cardiac glycosides, as including the calcium-dependant activation of caspases [34], inhibition of Hypoxia Inducible Factor-1α (HIF1α) synthesis, and interference with signal transduction pathways such as protein kinase Src, Epidermal Growth Factor Receptor (EGFR) or p21[17]. Because most GBM express EGFR [5] and since HIF1α is a marker gene upregulated upon hypoxia in GBM [35], proscillaridin A likely influences GBM tumor growth *via* various mechanisms.

We have shown that, *in vitro*, proscillaridin A exerts a dose-dependent cytotoxicity through both an increase in GBM cell death and a G_2_/M phase arrest. Cellular heterogeneity is a hallmark of GBM. In particular stem-like cells confer chemoresistance and are considered as the main brake to GBM therapy efficacy [7–11]. Our results showed for the first time that proscillaridin A impaired GBM stem self-renewal capacity and could be considered as a promising candidate for GBM treatment. In accordance, the number of Ki-67 expressing cells decreased in xenografts after proscillaridin A administration. We also learnt from *in vivo* studies that proscillaridin A induced a differentiation of GBM cells, as shown by the increase in GFAP expressing cells and accumulation of gliofilaments. Treatment also induced cell detachment *in vitro* and increased size of intercellular space *in vivo*. These results are in agreement with previous results reporting that ouabain, another cardiac glycoside, induced cell detachment of MDCK cells and that cells treated with ouabain accumulated in the G_2_/M phase in which they were more sensitive to radiation [36].

As a result of its cytotoxic properties, proscillaridin A was also shown here to be efficient to slow down the disease progression *in vivo*. Indeed, treatment inhibited tumor xenograft growth and increased the overall health and survival of *nude* mice implanted with GBM cell lines. These effects were measured in tumors obtained from two different cell lines and were observed successfully for intracranial xenografts whereas they were less obvious for subcutaneous xenografts.

To conclude, we have shown for the first time that proscillaridin A, known as a cardiac glycoside, also acts as a new therapeutic drug in human GBM. This study supports the proscillaridin A as a potent candidate for drug repositioning with advantages of toxicity and pharmacokinetics profiles already known as well as time- and cost-saving [37, 38]. To finish, further investigations to determine its underlying mechanisms as well as its place among the therapeutic strategy in GBM are warranted.

## METHODS

### Cell lines

GBM6 and GBM9 stem cell lines, established at our laboratory from 2 different human GBM tumor samples, exhibited features reminiscent of the clinical characteristics of the original tumors respectively [11, 12]. These cells were maintained and grown as floating spheres in serum-free medium supplemented with EGF and bFGF as previously described. When necessary, cells were grown in the same medium on 10 μg/ml poly-DL-ornithin. Under these conditions, they were able to adhere to the plastic without differentiating [11]. U87-MG and U251-MG (American Type Culture Collection, Rockville, MD, USA) were cultured as monolayers in Dubelcco's modified Eagle's medium (DMEM, Life technologies, Saint Aubin, France) supplemented with 10% fetal calf serum, 50 U/ml penicillin and 50 μg/ml streptomycin, at 37°C in a humidified atmosphere of 5% CO2 and 95% air.

### Tumor samples

Primary human GBM samples were collected at Assistance Publique – Hôpitaux de Marseille (AP-HM) and frozen samples were stored and provided by the AP-HM tumor bank (authorization number AC-2013-1786). Tumor specimens were obtained after written consent and according to a protocol approved by the local institutional review board and ethics committee. GBM were diagnosed according to the 2007 WHO classification [1]. Histological review (DFB) confirmed the neoplastic nature of the tissue containing at least 60% of tumor tissue and less than 40% of necrosis.

### Prestwick chemical library® screening: analysis of migration and proliferation of GBM6 and GBM9 cell lines *in vitro*

The Prestwick chemical library^®^ (Prestwick Chemical, Illkirch, France) contains 1120 molecules, 100% approved drugs (FDA, EMA and other agencies) and selected for their safety in humans. The 1120 molecules, all dissolved in DMSO and then diluted in sterile H_2_O to 100 μM, were screened in duplicate using scratch assay and flash-cytometer acquisitions [11]. Both cell migration and proliferation of GBM6 and GBM9 cells were measured after 3 days in culture on 96-well black plates (Greiner Bio-One, Courtaboeuf, France) coated with poly-DL-ornithin. Analyses were performed using MetaMorph® Software. Molecules were first used at a concentration of 2 μM. Only molecules reducing significantly the migration and proliferation of both GBM6 and GBM9 cells at 2 μM were analyzed at 0.1 μM and 0.05 μM.

### Gene expression analysis

Total RNA was isolated using TRI Reagent (Sigma-Aldrich, Paris, France) from GBM human samples (n = 26), GBM6 (n = 9 samples, passages 75, 78, 79, 80, 82, 83, 84, 85 and 86) and GBM9 (n = 7 samples, passages 80, 81, 82, 83, 84, 85 and 86) cell lines, cultured as floating spheres with serum-free medium supplemented with EGF and bFGF, U87-MG (n = 6 samples, passages 149, 150, 151, 154, 165, 167) and U251-MG (n = 5 samples, passages 56, 58, 67, 69, 72) adherent cell lines, cultured with standard conditions. RNA was analyzed on the spectrophotometer Nanodrop and Agilent 2100 bioanalyzer (Agilent Technologies, Massy, France). Before use, RNA samples were treated with 1U ribonuclease-free deoxyribonuclease (Roche Applied Science, Meylan, France) at 37°C for 20 min. One μg of total RNA was used for reverse transcription using random hexamers and Superscript II reverse transcriptase as recommended by the manufacturer (Invitrogen Life Technologies, Cergy Pontoise, France). The *ATP1A1* transcript was analyzed by RT-qPCR using a LightCycler^®^ 480 and the LightCycler 480 SYBRGreen I Master (Roche Applied Science, Meylan, France). Ribosomal *18S* and glyceraldehyde-3-phosphate-dehydrogenase (*GAPDH*) were used as reference genes. Forward and reverse primers for each transcript were designed using Primer express 3.0 software and are listed below: *ATP1A1*: 5′-tcgtcagtatcgtggtggtg-3′, 5′-gggcagtaggaaaggaaagc-3′; *18S*: 5′-ctaccacatccaaggaaggca-3′, 5′-tttttcgtcactacctccccg-3′; *GAPDH*: 5′-caaattccatggcaccgtc-3′, 5′-cccactygattttggaggga-3′. PCR conditions were as follows: 5 min at 95°C, followed by 45 cycles of 15s at 95°C and 30s at 65°C, 67°C and 65°C for *ATP1A1, 18S* and *GAPDH* respectively. Efficiencies were calculated from the given slopes in the LightCycler 480 software using serial dilutions of human total RNA reference (Stratagene, Massy, France). The relative expression ratio of the target gene transcript and reference gene transcripts was calculated as ddCt as previously described [39]. Quantifications were performed in triplicate for each sample. Results were expressed in arbitrary unit, as median and interquartile range (IQR).

### Viability assay

Proscillaridin A (Sigma-Aldrich, Saint-Quentin Fallavier, France) was dissolved at 18,8 mM in cell culture-grade DMSO at a maximum concentration of 0,5% and stored at −20°C until use. Cytotoxic effect of proscillaridin A on GBM6, GBM9, U87-MG and U251-MG cell lines was evaluated by assessing cell metabolic capacity, which reflects viability, using the MTT method (3-(4,5-dimethylthiazol-2yl)-diphenyl tetrazolium bromide, Sigma-Aldrich). GBM6 and GBM9 were seeded in previously poly-DL-ornithin-coated 96-well plates (7500 cells/well). U87-MG and U251-MG were seeded in 96-well plates (7500 cells/well). After 24h, cells were treated with serial concentrations of proscillaridin A (0; 0,01; 0,1; 0,5; 1; 5; 10; 50; 100; 500; 1000 nM) in 200 μl of cell-specific media per well. After 72h treatment, 20 μl of MTT reagent were added to each well and plates were incubated for 3h at 37°C. The reduced formazan was dissolved in DMSO and absorbance was measured at 562 nm with an Elx800 microplate reader (Bio-Tek, France). The cell viability was expressed as a percentage as compared to DMSO control cells. A concentration of proscillaridin A required to inhibit 50% of cell viability after 72h of drug incubation (IC_50_) was deduced for each cell line using CalcuSyn software. The experiments were carried out 3 times in triplicate.

### Cell morphology analysis

Briefly, 150 000 cells/well were seeded in a previously poly-DL-ornithin-coated 6-well plate for GBM6 and GBM9 cell lines whereas U87-MG and U251-MG cell lines were seeded in 6-well plates. Cells were treated with IC_50_ concentration of proscillaridin A or DMSO for 72h. Proscillaridin A impact on cell morphology was examined using an inverted light microscope (Leica DMI 4000 B).

### DNA fragmentation and cell cycle analysis

DNA fragmentation and cell cycle analyses using flow cytometry (FACS Calibur, BD Biosciences, Le Pont de Claix, France) were performed by propidium iodide (PI) staining. GBM cells were seeded at 150 000 cells/well into previously poly-DL-ornithin-coated 6-well plates (GBM6 and GBM9) or not (U87-MG and U251-MG) and treated with IC_50_ concentration of proscillaridin A or DMSO for 72h. Cells were trypsinized and resuspended into 0.1% trinatriumcitrat-dihydrat pH 7,4, 0,1 % triton X10 and 100 μg/ml PI for at least 2h on ice. A total of 10 000 events were counted for each sample. Results were harvested with the CellQuest Pro Software (BD Biosciences) and analyzed using FlowJo software choosing the Dean-Jet-Fox model analysis.

### Self-renewal analysis

Effect of proscillaridin A on self-renewal capacity on GBM stem cells was evaluated by limiting dilution [40]. GBM6 and GBM9 stem cells were seeded in previously poly-DL-ornithin coated 6-well plates (150 000 cells/well) in the presence of 1.5 mL of serum-free medium supplement with EGF and bFGF. After 24h, cells were treated with IC_50_ concentration of proscillaridin A or DMSO for 72h. At the end of treatment, the supernatant was removed, cells were harvested, dissociated into single cells and plated in 96-well plates (1–5 cells/well) with serum-free medium supplement with EGF and bFGF. Eight days later, the spheres were counted. The sphere formation efficacy was calculated as the number of spheres formed divided by the original number of cells seeded. Three experiments were performed in duplicate for each cell line. In addition, 30 000 treated dissociated cells were also plated in a dish to assess spheres formation and were photographed 8 days later under an inverted light microscope (Leica DMI 4000B).

### Subcutaneous injections

All experimental procedures and animal care were carried out according to a protocol approved by the local institutional review board and ethics committee and according to national regulations. Five hundred thousand of GBM6 cells (passage 72) were subcutaneously transplanted into the right flank of 6-week-old athymic *nude* mice (Harlan France, Gannat, France); a total of 10 mice were injected. Usually, tumors were apparent from 3 to 5 weeks after injection (as determined using a vernier caliper and the standard formula tumor volume = (thickness x length x width) x π / 6). Same procedure was conducted with 500 000 U87-MG cells (n = 29 mice). In that case, tumors were apparent from 1 to 2 weeks after injection. As soon as the subcutaneous tumors reached 2-4 mm^3^ volume, the mice were randomized into 2 equivalent groups and given on intraperitoneal (i.p.) injection (200 μl bolus) 5 days a week of either proscillaridin A (7 mg/kg body weight diluted in phosphate buffered saline (PBS)) or vehicle (DMSO diluted in PBS). The tumor size and general clinical status were recorded every 4 days. After 3 weeks of treatment, mice were euthanized, tumors excised and rinsed in PBS. A portion of 1 mm^3^ was fixed in glutaraldehyde 2.5%. Then the remaining of the tumor was formalin-fixed and paraffin-embedded for histology analysis.

### Intracranial injections

Six-week-old athymic *nude* mice were anesthetized and 50 000 cells of GBM6 or U87-MG cells were stereotactically injected in the corpus callosum (1 mm anterior to bregma, −1mm lateral and −2 mm in deep of the cortex surface) as previously described [11]. Animals were observed until they fully recovered. A total of 21 mice were injected for GBM6 cell line and 17 mice were injected for U87-MG cell line. They were randomized into 2 groups. For GBM6, 11 mice were given on i.p. injection (200 μl bolus) 5 days a week of proscillaridin A (7 mg/kg body weight) and 10 mice were treated with the same volume of vehicle. For U87-MG, 10 mice were treated with proscillaridin A and 7 mice were treated with vehicle. According to tumor graft delay, i.p. treatment was started 2 weeks after U87-MG cells injection and 2 months after GBM6 cells injection. The body weight and clinical status of mice were recorded every 2 days. Mice were euthanized when exhibited more than 20% reduction from initial body weight or significant neurological deficit. Brains were extracted, fixed in formalin and paraffin-embedded according to standard procedures.

### Histology and immunohistochemical analysis

For each subcutaneous tumor, 4 μm thick paraffin sections were prepared and stained with hematoxylin and eosin for histological study. All brains were sectioned and stained with hematoxylin and eosin to attest to the presence of a tumor. Conventional immunohistochemistries for Ki-67 (30–9, Ventana Medical Systems, Illkirch, France) and GFAP (EP672Y, Ventana Medical Systems) were performed using the Benchmark XT automate (Ventana Medical Systems) according to the manufacturer's instructions.

### Transmission electron microscopy

Ultrastructural study was performed using transmission electron microscopy. Tissue samples fixed in glutaraldehyde 2.5% and post-fixed in osmium tetroxyde 2%, were embedded with epoxy resine in beam capsule. Polymerization was performed at 56°C. Ultra-thin sections (50 to 80 nm thick) were collected on copper grids then counterstained 15 min with uranyl acetate 5% and 5 min with lead citrate 0.1%. When dried, the grids were analyzed with JEM 1400 microscope (JEOL, USA).

### Statistical analyses

The non-parametric Wilcoxon test was used to compare the effect of emetine dihydrochloride, proscillaridin A and strophantidin on GBM6 and GBM9 cell migration and proliferation rates and to analyze cell cycle distribution differences between proscillaridin A treated cell lines and DMSO control cell lines. Cell self-renewal capacity was analyzed by the non-parametric Mann-Whitney test. To compare the mean tumor volume variations in the two subcutaneously transplanted groups (vehicle *vs* proscillaridin A) we performed the analysis of variance (ANOVA) test, and after confirmation of normality, the means were compared by the ANOVA post-hoc Tukey test. Overall survival curves of intracranial xenografted mice were obtained according to the Kaplan-Meier method and compared using the Log-rank test. All statistical tests were two-sided and the threshold for statistical significance was *p*<0.05. Wilcoxon, Mann-Whitney and ANOVA tests were conducted using the XLSTAT 2013 software (Addinsoft, Paris, France) whereas survival analyses were made using the GraphPad Prism4 software (GraphPad, Inc.)
